# The Efficacy of Virtual Reality Game Preparation for Children Scheduled for Magnetic Resonance Imaging Procedures (IMAGINE): Protocol for a Randomized Controlled Trial

**DOI:** 10.2196/30616

**Published:** 2022-06-13

**Authors:** Sylvie Le May, Christine Genest, Nicole Hung, Maxime Francoeur, Estelle Guingo, Julie Paquette, Olivier Fortin, Stéphane Guay

**Affiliations:** 1 Research Center Centre Hospitalier Universitaire Sainte-Justine Montreal, QC Canada; 2 Faculty of Nursing Université de Montréal Montreal, QC Canada; 3 Trauma Studies Centre Institut Universitaire en Santé Mentale de Montréal Centre Intégré Universitaire de Santé et de Services Sociaux de l’Est-de-l’Île de Montréal Montreal, QC Canada; 4 Faculty of Medicine Université de Montréal Montreal, QC Canada; 5 Université du Québec en Abitibi-Témiscamingue Rouyn-Noranda, QC Canada; 6 School of Criminology Université de Montréal Montreal, QC Canada

**Keywords:** virtual reality, children, video games, magnetic resonance imaging, anxiety, pediatrics, patient collaboration, patient preparation, biofeedback

## Abstract

**Background:**

It is known that magnetic resonance imaging (MRI) procedures generate fear and anxiety. Children may become restless during scanning, which results in movement artifacts requiring the MRI procedure to be repeated with sedation. Few studies seem to have looked at the effect of immersive virtual reality (IVR) on anxiety in children scheduled for MRI scans and how to identify which children are more responsive.

**Objective:**

The aims of this study are 3-fold: develop an algorithm of predictability based on biofeedback, address feasibility and acceptability of preprocedural IVR game preparation for anxiety management during MRI procedures, and examine the efficacy of IVR game preparation compared with usual care for the management of procedural anxiety during MRI scans.

**Methods:**

This study will have 2 phases. We will first conduct a field test with 10 participants aged 7 to 17 years to develop a predictive algorithm for biofeedback solution and to address the feasibility and acceptability of the research. After the field test, a randomized controlled trial will be completed using a parallel design with 2 groups: an experimental group (preprocedural IVR game preparation) and a usual care group (standard care as per the radiology department’s protocol) in an equal ratio of 49 participants per group for 98 participants. Recruitment will be carried out at a hospital in Quebec, Canada. The experimental group will receive a preprocedural IVR game preparation (IMAGINE) that offers an immersive simulation of the MRI scan. Participants will complete a questionnaire to assess the acceptability, feasibility, and incidence of side effects related to the intervention and the biofeedback device. Data collected will include sociodemographic and clinical characteristics as well as measures of procedure-related anxiety with the French-Canadian version of the State-Trait Anxiety Inventory for Children (score 1-3) and the Children’s Fear Scale (score 0-4). Physiological signs will be noted and include heart rate, skin conductance, hand temperature, and muscle tension. Measures of the level of satisfaction of health care professionals, parents, and participants will also be collected. Analyses will be carried out according to the intention-to-treat principle, with a Cronbach α significance level of .05.

**Results:**

As of May 10, 2022, no participant was enrolled in the clinical trial. The data collection time frame is projected to be between April 1, 2022, and March 31, 2023. Findings will be disseminated through peer-reviewed publications.

**Conclusions:**

Our study provides an alternative method for anxiety management to better prepare patients for an awake MRI procedure. The biofeedback will help predict which children are more responsive to this type of intervention. This study will guide future medical practice by providing evidence-based knowledge on a nonpharmacological therapeutic modality for anxiety management in children scheduled for an MRI scan.

**Trial Registration:**

ClinicalTrials.gov NCT04988516; https://clinicaltrials.gov/ct2/show/NCT04988516

**International Registered Report Identifier (IRRID):**

PRR1-10.2196/30616

## Introduction

### Background

Magnetic resonance imaging (MRI) is a technique that is considered noninvasive and safe because it does not use any radiation or x-rays unlike positron emission tomography scans or computed tomography scans. MRI technology, instead, uses a magnetic field to generate images of the tissues and organs. For MRI procedures to work as intended, the patient must remain still while lying down within a confined space for a certain amount of time.

The scan environment may be a source of anxiety for some patients. This can be due to the claustrophobic nature of a narrow space. MRI procedures have been known, for almost 40 years, to generate fear and anxiety caused by claustrophobia [1]. In addition, MRI scanners generate loud clicking sounds while running. These loud acoustic noises may be as loud as 100 dB, the equivalent of a snowmobile next to the patient [2]. Not surprisingly, up to 30% of the patients undergoing MRI procedures had anxiety-related reactions of varying degree of intensity [3]. Interviews performed with younger children and their parents revealed that MRI procedures caused anxiety in children because of their size, design, and sound [4]. Hence, this environment is difficult to tolerate, especially for children. Often, anxious children may become restless during the examination, leading to uncontrolled movements resulting in undiagnostic images. As a consequence, this may result in a premature termination of the procedure itself, requiring the examination to be repeated ulteriorly, thus causing subsequent episodes of anxiety [4-6].

As a result, it is becoming common practice in radiology departments in many hospitals to require conscious sedation, as the frequency of repeated MRI scans is higher in the pediatric population [5-7]. However, sedation is not without any risk or consequences. Emerging evidence suggests that sedation in children might have long-term neurocognitive side effects, in addition to the short-term procedure-related risks [8]. As some authors also pointed out, its use is also related to an increased amount of fear in children and their parents and will require extended hospital stays for monitoring, adding to the cost burden [9].

Similar to any medication, there is always a risk of adverse reactions. Therefore, several efforts have been directed into the development of nonpharmacological methods to reduce fear and anxiety in children requiring MRI scans. Many interventions ranging from music and artwork to video games have been used and deemed useful to relieve anxiety in children during an MRI examination [10,11]. Interventions performed before the scan have also been investigated. Among these, preprocedural patient education has been shown to decrease anxiety and enhance comprehension of MRI examinations, which in turn, can serve to increase patient collaboration [12]. However, different education material can have different effects on reducing anxiety levels [13]. Mock MRI scanners, which involve using a full-size replica scanner for a 5-minute training session to lie still, have been used to help explain to children what the procedure involves and what to expect in an age-appropriate manner. This preparation has been reported to reduce MRI examination–related procedural anxiety, rate of motion artifacts, need for sedation, overall duration of the study, as well as a decrease in heart rate during the procedure [14,15]. However, a shortcoming of the use of mock replicas is that there is limited availability in hospitals, because the mock MRI machine would require a room to be stored in, as well as additional resources, including staff and time, to organize these sessions.

Physical mock sessions before MRI scans have shown promising results. Therefore, virtual reality (VR) used to replicate an MRI environment can also be used as patient preparation [16]. Preprocedural VR education has been studied in different medical procedures, including chest radiography, dental procedures, anesthesia, and surgeries [5,17,18]. These studies show that VR preparation helps improve procedural experience among pediatric patients by reducing anxiety, distress, and procedure time while increasing parents’ satisfaction. VR is a novel technology gaining popularity in pediatric hospitals worldwide for a variety of reasons. It is a distraction method that has proven effective in reducing pain and anxiety in children in different settings such as phlebotomy, wound care, chemotherapy, dental procedure, and bone pins removal [19-23].

To the best of our knowledge, very few studies have looked at the effect of VR on anxiety in children scheduled for an MRI scan specifically and how this intervention could help identify which children are more responsive to VR. As VR technology is becoming progressively more accessible, we believe that incorporating a VR preparation tool ahead of time to familiarize children before the MRI procedure would help decrease anxiety; increase patient collaboration; decrease the need for sedation; and improve satisfaction for the patient, family, and health care professionals.

### Aims of the Study

The aims of this study are 3-fold: (1) develop an algorithm of predictability based on biofeedback, (2) address feasibility and acceptability of a preprocedural immersive VR (IVR) game preparation before an MRI procedure for children’s anxiety (field test phase), and (3) examine the efficacy of a preprocedural IVR game preparation compared with usual care for the management of procedural anxiety in children undergoing an MRI examination (by conducting a randomized controlled trial [RCT]).

### Hypothesis (for Scientific Validation)

We believe that an IVR intervention in the form of an interaction-enabled video game to prepare participants before an MRI examination is easy to use and could help decrease MRI examination–related procedural anxiety in children aged 7 to 17 years. We believe that a patient who follows instructions well, without any signs of anxiety detected by physiological parameters, will have better results in the MRI procedure than a patient who has difficulty following instructions or who shows signs of anxiety through their physiological parameters.

### Objectives

The primary research question concerns whether preprocedural interaction-enabled IVR game preparation will decrease MRI examination–related procedural anxiety for children undergoing an MRI procedure. The secondary objectives of the scientific clinical validation phases are as follows:

1. To determine whether preprocedural IVR game preparation is a feasible and acceptable nonpharmacological method to decrease MRI examination–related procedural anxiety.
2. To determine whether children experiencing preprocedural IVR game preparation will have a slower heart rate before and during the MRI procedure than children not exposed to the IVR game preparation.
3. To determine whether children experiencing preprocedural IVR game preparation will require lower need for sedation than children not exposed to the IVR game preparation before an MRI examination.
4. To determine whether children experiencing preprocedural IVR game preparation will require rescheduling of the examination less often than children not exposed to the IVR game preparation before an MRI examination.
5. To evaluate the occurrence of side effects with preprocedural IVR game preparation in comparison with children not exposed to the IVR game preparation before an MRI examination.
6. To compare satisfaction levels of health care professionals between preprocedural IVR game preparation and usual care groups.
7. To compare satisfaction levels of children and parents between preprocedural IVR game preparation and usual care groups.
8. To compare overall procedure time required for an MRI examination between preprocedural IVR game preparation and usual care groups.
9. To develop a predictability algorithm that will help identify which children will have better results in the MRI procedure after the IVR game preparation.

## Methods

### Design

This study will have 2 phases. We will first conduct a field test with 10% (10/100) of the total sample size calculated to initiate the development of a predictive algorithm for biofeedback solution requiring actual participants and to address the feasibility and acceptability of the VR intervention and research process. The field test phase will follow the steps indicated in this protocol. Any changes needed will be made between the end of the field test and the start of the RCT. No changes will be made once the RCT starts. After the field test, we will proceed to a scientific clinical validation based on an RCT design using a parallel design with 2 groups: an experimental group (preprocedural IVR game preparation) and a usual care group (standard care as per the protocol of the radiology department) in an equal ratio of 49 participants per group for a total of 98 participants, including a correction for an attrition rate of 24.9% (48/193) that allows for delays and repetitions of procedures, established according to the 2020 radiology records at the study setting.

### Sample and Setting

Recruitment will be carried out at Centre Intégré Universitaire de Santé et de Services Sociaux de l’Est de l’Île de Montréal, Quebec, Canada, a general care hospital with a pediatric unit and services. Participants will be identified through the radiology information system as having an appointment for an upcoming MRI procedure. A research nurse will be notified by the radiology technologist and will proceed to contact the parents for recruitment ahead of time before their arrival at the radiology department. On the day of the appointment, parents will be approached to sign the consent if they still agree to the study. Assent from the child will also be obtained on the same day. Of note, owing to the COVID-19 sanitary crisis, availability of recruiting personnel, and the difficulty of movement between units and departments, recruitment will be limited to the radiology department. According to the statistics in 2020, a total of 145 MRI procedures were prescribed for children aged 7 to 17 years at this setting, but in reality, 193 procedures were carried out because of delays and repetitions of procedures. Thus, an attrition rate of 24.9% (48/193) was considered in the calculation of the sample size.

### Inclusion Criteria

Children and their parents will be invited to participate in the study if they meet the following inclusion criteria: (1) aged 7 to 17 years; (2) required to undergo an MRI procedure; and (3) accompanied by a consenting parent or legal guardian who can understand, read, and write either French or English.

### Exclusion Criteria

Participants will be excluded from the study if they (1) are diagnosed with epilepsy or any other condition preventing them from playing a VR game or (2) cannot tolerate a sitting or semiupright sitting position (Fowler position) during the preparation because the VR gear requires an angle of at least 30° for head tracking. Participants who received anxiolytics (eg, benzodiazepines) in the last 24 hours before the MRI procedure will not be excluded, but the names and dosages of the medication and time of administration will be documented in the data collection form.

### Interventions

Standard preparation as per the radiology department’s protocol will serve as the control (usual care) group.

Preprocedural IVR game preparation will serve as the intervention group.

### Control Treatment

The usual care group will only receive the standard preparation, which comprises of an explanation of the MRI procedure given by the radiology technician as per the radiology department’s protocol. The research nurse will be able to complete any information should they find it incomplete or substandard.

### Experimental Treatment

The preprocedural IVR game preparation (IMAGINE) was developed by Paperplane Therapeutics (an intervention development team). IMAGINE is an IVR simulation game intended for young patients aged 7 to 17 years who will undergo an MRI examination. It aims to reduce anxiety and phobic reactions of young patients and to better prepare them for this examination. As the game is a preparation, it is a no-success game and is independent of the child’s ability and previous experience with video games. IMAGINE is designed to be supported by stand-alone VR headsets such as Oculus Quest and Pico Neo II. This preprocedural IVR game preparation offers an immersive and fun simulation that aims to desensitize young patients to MRI procedures and transform an anxiety-inducing experience into a fun play session. From a game point of view, the patient takes a seat aboard a spaceship. The child will have to complete various quests to activate the MRI machine, which is portrayed as a very useful device for the crew members as it will enable them to promulgate the appropriate medical care. The participant will learn about the main principles of MRI procedures and experience a very realistic simulation of the MRI examination. It is developed with personalized care content tailored to children to maximize the feeling of immersion and minimize cybersickness. The preprocedural IVR game preparation is approved by a team of health care professionals in pediatric care.

IMAGINE includes an interaction-enabled VR replica of the inside and outside environment of the scanner, including visual and audio effects of an actual MRI examination, to allow the child to be exposed to the MRI journey in a fun and interaction-enabled way before the examination using the Pico Neo II VR Helmet. This will allow children to prepare for the MRI scanner by enabling them to experience the process of undergoing an MRI examination beforehand, thereby improving their understanding of upcoming events and eventually prevent, decrease, or control preprocedural and MRI examination–related procedural anxiety. The simulation already contains a cursor in the center of the patient’s field of view and follows the head movements. A round target inside the replica of the MRI device tells the patient how to place their head in the neutral position and gives positive feedback when done. During the simulation, head movement information will be extracted from the positioning cameras, accelerometer, and gyroscopes already available in the headset. A VR tour session will be available in the waiting area for patients and parents before the MRI procedure. An approved and validated device (such as Thought Technology [24]) will also be used during the simulation to continuously record biofeedback data such as heart rate, skin conductance, hand temperature, and muscle tension. We will also ask participants to undertake the IVR experience and complete a questionnaire to assess the acceptability, feasibility, and incidence of side effects related to the intervention and the biofeedback device. In the likely event of an episode of dizziness, nausea, or vomiting, the child will be provided with proper care in accordance with the unit’s protocol and will immediately be removed from the IVR. According to the results obtained, we will adjust the protocol and then proceed to the scientific clinical validation to evaluate the efficacy of this preprocedural IVR game preparation in the management of MRI examination–related procedural anxiety in children and to develop a predictability algorithm. The VR intervention will last for 15 to 20 minutes, which is considered an adequate amount of time to maintain a child’s attention and allow for a complete experience of the MRI procedure, including the room’s environment and audiovisual stimuli. While the child plays with the interaction-enabled IVR game preparation, there is also the option to allow parents to observe what the child is viewing through their headgear on a separate screen. Unfortunately, due to lack of physical space within the preparation room, only 1 parent will be able to assist during the VR session.

### Study Time Points

Sociodemographic and clinical characteristics will be assessed in the waiting room to establish baseline at T0. Measures of procedure-related anxiety with the State-Trait Anxiety Inventory for Children, French-Canadian version (STAIC-F; score 1-3) and also the Children’s Fear Scale (CFS; score 0-4) will be taken before the intervention (T0), immediately after the intervention (T1), and after the MRI procedure (T2). A measure of the level of satisfaction of health care professionals, parents, and participants through a questionnaire developed and pretested by the team will also be collected at T2. Physiological signs such as heart rate, skin conductance, hand temperature, and muscle tension through an electromyogram will be collected throughout the simulation. Data will be collected on the occurrence of side effects throughout the study. Clinical monitoring will be performed by an independent nurse from the research team.

### Ethics Approval

Ethics approval (2022-2554) was obtained in March 2022 by the Ethics Board of the CIUSSS de l’Est-de-l’Île-de-Montréal.

### Measures and Outcomes

#### Sociodemographic and Clinical Questionnaire

Demographic and clinical characteristics will be assessed with parent reports filled out by the research nurse using the case report form and will include data on age, sex, ethnicity, reason for MRI imaging study, and history of imaging studies, including MRI, computed axial tomography scans, and x-ray examinations. Data such as name, dosage, and time of administration of anxiolytics taken 24 hours before the MRI examination will be collected. The questionnaire will also include a section describing the context of the procedure including adherence to the intervention, total procedure time, use of other nonpharmacological interventions, and the occurrence of side effects.

#### Criteria for Feasibility Assessment

The field test phase will be assessed for feasibility using the CONSORT (Consolidated Standards of Reporting Trials) checklist for pilot studies to determine whether proceeding to the full study is possible. Any modification needed to the collection process will be carried out at this stage. No modification will be carried out once the RCT starts.

#### Primary Outcome

The primary outcome will be the mean difference in State anxiety at T2 for both study groups as measured by the S scale of the STAIC-F.

#### Measures of Primary Outcomes

The level of State anxiety (primary outcome) will be assessed using the State Scale of the STAIC-F questionnaire [25,26]. The STAIC-F (Multimedia Appendix 1 [25,26]) is a self-report instrument inspired by the State-Trait theory extended by Spielberger [27] that measures a momentary state of anxiety (state) and a stable tendency to experience anxiety (trait). The same author obtained internal consistency of coefficients of 0.87 for girls and 0.82 for boys [25]. The first 20 items constitute the situational (State) anxiety scale. The response “almost never” is graded 1, the response “sometimes” is graded 2, and the response “always” is graded 3, for a total score varying between 20 and 60 [26]. The next 20 items deal with Trait anxiety. The quotation is identical. A table is provided, which presents the average scores by age and sex. A score can be considered high when the child is ≥1 SD from the mean. It is typically completed in 8 to 12 minutes for initial evaluation, and subsequent evaluations typically require 5 to 7 minutes. The scale was developed specifically for use for children aged 9 to 12 years but can be used in younger children if the scale items are read out loud to them, as well as for older children [25]. Participants will rate their State-Trait anxiety with the French-Canadian version 40-item questionnaire (STAIC-F) in the waiting room (T0). The first 20 items, which correspond to situational anxiety, will be reassessed after the intervention (T1) and after the MRI examination (T2). Psychometric studies of the STAIC-F show excellent internal consistency coefficients of the State-Trait Anxiety Inventory for Children (STAIC) scales, with Cronbach α values of .89 and .88, respectively, and the test-retest reliabilities after a 6-month period were also similar to those of the original version, and the concurrent validity, assessed by the correlation with the Revised Children’s Manifest Anxiety Scale, was also found to be good [26]. A study investigated MRI examination–related anxiety in children using the English shortened-version of the STAIC scale for children aged 8 to 15 years [28], and 2 other studies examined MRI examination–related anxiety using the standard English STAIC scale for its robust psychometric properties for children aged 8 to 17 years and 12 to 18 years [29,30]. Considering the French-speaking population targeted in this study, the psychometrically sound properties of the French-Canadian version, and previous studies using the STAIC questionnaire to evaluate MRI examination–related procedural anxiety, we believe that it is justified to use this questionnaire in this study for children aged 7 to 17 years to evaluate their levels of anxiety.

The level of anxiety will be also assessed using the CFS [31] at baseline before the preprocedural IVR game preparation or usual care intervention (T0), immediately after the intervention (T1), and after the MRI procedure (T2; Multimedia Appendix 2). The CFS is a self-reported measure of anxiety adapted from the Adult Faces Anxiety Scale [32] for specific use in children undergoing painful experiences. It consists of 5 faces ranging from 0 (*no fear or anxiety*) through 4 (*extremely fearful or anxious*). The child is asked to indicate which face shows best how they felt during the procedure. Test-retest reliability has been established with children aged 5 to 10 years during painful procedure (*r_s_*=0.76) and concurrent validity with the Children Anxiety and Pain Scale (*r_s_*=0.73) [33]. We chose the CFS because of its psychometric qualities and because it is a 1-item self-measure of anxiety and fear. Moreover, *“*although fear tends to decrease with age in general, medical fears may be an exception*”* [34]. In addition, “age-related differences in fear ratings have not been found*”* [35]. Furthermore, this scale has been used with children aged 8 to 18 years for procedural fear related to venipuncture [36] and children aged 11 to 13 years for fear of, and anxiety regarding, vaccination [37].

#### Secondary Outcomes

The secondary outcomes were (1) mean difference in the sense of presence and engagement (immersion) into the VR game between groups (T1), (2) the presence of head deviation during the intervention, (3) changes in physiological signs through biofeedback during the intervention, (4) satisfaction levels of parent (T2), (5) satisfaction levels of children (T2), (6) satisfaction levels of health care professionals (T2), (7) occurrence of side effects, (8) mean difference in total procedure time and frequency of rescheduling MRI examinations between groups, and (9) mean difference in Trait anxiety levels between groups.

#### Measures of Secondary Outcomes

The secondary outcomes were measured as follows:

1. The sense of presence and engagement (immersion) in the VR game will be assessed using the Graphic Rating Scale (GRS) [38-40], a 7-item Likert-type scale tailored for VR interventions. Convergent validity among pairs of scores of the GRS with children aged 8 to 17 years was *r*=0.84 and test-retest reliability was 0.91 [40].
2. The head deviation during the simulation will be measured continuously for the entire duration of the intervention through the positioning cameras, accelerometer, and gyroscopes in the headset.
3. Physiological signs and biofeedback (heart rate, skin conductance, hand temperature, and muscle tension via an electromyogram) will be measured using the biofeedback device that will continuously capture and record physiological signs. The research nurse will note the heart rate measures at T0; immediately after T1; and at 1, 5, 10, and 15 minutes of the MRI procedure. Increased heart rates may indicate physiological arousal or may be a consequence of “positive stress” [41].
4. Satisfaction levels of the parent will be assessed using a 0 to 10 numerical scale to answer the following question as recommended by Pediatric Initiative on Methods, Measurement, and Pain Assessment in Clinical Trials (PedIMMPACT) [42]: “Considering anxiety relief, side effects, and emotional recovery, how satisfied were you with the treatments your child received for anxiety?”
5. Satisfaction level of the children will be assessed using a 0-to-10 numerical scale to answer the following questions: “How fun is the game?” “Did the game help you feel less scared during the MRI examination?” and “Will you recommend this game to other children who have to go through the same examination as you?”
6. Satisfaction level of the health care professional will be assessed using a tailored questionnaire with a 4-choice response scale from strongly agree to strongly disagree for 7 items related to their level of satisfaction with the intervention and its effects on the procedure.
7. Occurrence of side effects will be assessed and documented from enrollment until discharge.
8. Procedural time and number of rescheduled MRI examinations will be noted in the clinical questionnaire.
9. Trait anxiety level will be measured using the T scale of the STAIC-F.

### Sample Size and Statistical Analysis

#### Sample Size Consideration

Primary analysis will involve the comparison of 2 group means. In addition, no interim analysis will be conducted. Therefore, group sample sizes of 37 (ie, 74 in total) are necessary to achieve 80% power to reject the null hypothesis of equal means when the population mean difference for State anxiety score is 5 with an SD for both groups of 7.47 [28] and a significance level (Cronbach α) of 5% using a 2-tailed *t* test. The SD for both groups varied from 4.61 to 7.47 [28]. To be conservative, we chose 7.47. On the basis of data from the medical imaging registry, the attrition rate was approximately 24%. Assuming a similar attrition rate, 98 participants (49 per group) will be required. The sample size calculation was performed using PASS Software (version 12.0; NCSS Statistical Software).

#### Statistical Analysis

Analyses will be conducted using SAS statistical analysis software (version 9.4; SAS Institute Inc). Descriptive statistics will be conducted for sociodemographic and clinical variables and presented by treatment group.

##### Primary Outcome Analyses

An analysis of covariance (ANCOVA) adjusted for age, sex, baseline (T0) Trait anxiety score measurement, and baseline State anxiety score measurement will be used to assess the mean difference in State anxiety scores on the STAIC-State between the experimental and the control groups at T2. Analyses will be carried out according to the intention-to-treat principle, with a significance level (Cronbach α) of .05.

##### Secondary Outcome Analyses

An ANCOVA adjusted for age, sex, and baseline anxiety score measurement will be used to assess the mean difference in anxiety scores on the CFS, between the experimental and the control groups at T2. To assess the mean difference in the sense of presence in VR and engagement into the game (GRS), between the experimental and the control groups at T1, an ANCOVA adjusted for age and sex will also be conducted. We will use a linear mixed model to estimate the effect of the treatment on the changes in heart rate over all assessment time points. This analysis will be adjusted for age, sex, and baseline heart rate. Differences between arms for levels of satisfaction of parents, children, and health care professionals (T2) as well as the overall procedure time will be assessed using (2-tailed) *t* tests or nonparametric Mann-Whitney *U* tests if data are nonnormal. A chi-square test or Fisher exact test will be conducted to compare dichotomous variables including the occurrence of side effects, the number of rescheduled MRI examinations, and the use of sedation in each group.

Adverse events and serious adverse events (if any) will be reported using the Medical Dictionary for Regulatory Activities terminology, and their proportions will be compared between the groups.

To help develop the predictability algorithm, the head deviations and other physiological data will be analyzed. An algorithm based on those deviations will be developed prospectively as the study progresses to evaluate the success of the MRI procedure in the intervention context and offer useful predictability inputs in preparation for the real MRI examination. No existing algorithms specific to the study were found. The physiological data will also help create a time sequence that could be matched with the information that will be extracted from the intervention. At the end of the session, it will be possible to see if, for example, a movement of the patient is generally triggered by an increase in stress as captured by the sensors. The result of the examination will then be compared with the data obtained during the intervention. The team will attempt to determine which variables correlate with the real-life outcome. Our hypothesis is that a patient who can follow the instructions well without any signs of anxiety captured by physiological parameters will have better results in the MRI procedure than a patient who has difficulty following the instructions or who shows signs of anxiety expressed through their physiological parameters.

### Study Proceedings (Clinical Validation Phases)

Participants will be identified through the radiology information system as having an appointment for an upcoming MRI procedure. Either the physician prescribing the MRI examination or the radiologist will have indicated certain patients as not needing sedation during the MRI examination and thus identified as eligible for this study. The research nurse will be notified by the radiology technologist and will contact the parents for recruitment a few days before the procedure. On the day of the appointment, parents will be approached to sign the consent if they still agree to the study. The child’s assent will also be obtained on the same day. Participants are informed of their assignment to either the preprocedural IVR game preparation or usual care group after randomization. Before initiation of the procedure, the research nurse will explain the intervention to the patient and install the IVR game preparation and start the video game program. Baseline measurements of anxiety will be taken before the interventions in the waiting room (T0).

The research nurse will log in 10 minutes before the IVR video game preparation or usual care interventions to obtain the group allocation of the participant if they meet all eligibility criteria. Given the nature of the interventions, the participants and personnel cannot be blinded to the interventions. However, to minimize observer bias given the subjective measures of outcomes, parents and children will only be informed to which group they are assigned 10 minutes before the intervention. Data will be collected on paper forms and then the information will be transferred into REDCap (Research Electronic Data Capture; Vanderbilt University) by a research nurse. Data on game completion by the child will be recorded but no data will be collected on specific game inputs. Furthermore, although a separate screen allows research staff and parents to watch the child’s perspective, none of it will be recorded. The paper copies of the data forms will be kept locked. The data will be reviewed by a data manager to check for possible errors. The informed consent, assent, and baseline data collection will require approximately 10 minutes. After randomization, the IVR game preparation intervention will last for 15 to 20 minutes. Postintervention data collection will require approximately 10 minutes. In total, the duration of the intervention period, from recruitment to discharge, will be 30 to 40 minutes for each participant. Time has been allocated to the clinical workflow to allow for the research nurse to clean the VR technology before the arrival of each new patient.

Data collection forms of the participants will be stored under a double lock at one of the principal investigators’ office at the research center and will be kept for 7 years after the end of the trial. As this is an open-label clinical trial and no prior data suggest that any of the 2 interventions are associated with side effects, a data monitoring committee is not required. However, the occurrence of any side effects will be reported to the ethics committee. VR technology such as a head-mounted display (eg, Pico Neo II VR Helmet) has the potential to cause motion sickness, including discomfort, disorientation, and nausea. To overcome this issue, the development team specialized in immersive technology had previously developed a game that fully maximizes the experience of immersion and the sense of being in a virtual environment while minimizing motion sickness. During a previous pilot study using this VR game, no participant reported motion sickness symptoms while using IVR [43].

### Privacy and Confidentiality

A consent form will be presented by a research nurse to participants and their parents or legal guardian to clearly explain the use of the VR technology, its expected benefits and adverse effects, and any necessary information that may be collected for research purposes.

## Results

Recruitment began in April 2022. As of May 10, 2022, only 1 participant for the field test was recruited. No participant for the clinical trial has been recruited as yet. The data collection time frame is projected to be between April 1, 2022, and March 31, 2023. The study period will end in April 2024. The findings will be disseminated through peer-reviewed publications.

## Discussion

### Feasibility of the Study

The feasibility and acceptability of the research protocol will be addressed in the field test phase, which will be followed by a parallel design RCT with 2 groups. The development of a VR game preparation tool can help increase patient collaboration by distracting and calming children before entering the MRI examination room, which in turn will decrease the number of failed tests and repeat tests and hence increase the efficiency of the radiology department. We believe that the use of VR in the pre–MRI examination setting in the form of a fun and interaction-enabled video game can help reduce MRI examination–related anxiety in children by familiarizing them with the MRI environment. Very few studies in the field have investigated the effect of VR preparation for MRI scans for children, but the use of a VR-environment education tool before radiotherapy in adults has shown promising results such as improved education, reduced anxiety, and improved patient-reported satisfaction by improved comprehension of the medical procedure [12]. Other studies have also used VR exposure as a preparation tool for elective day-care surgery in children, and results suggest lower levels of anxiety, pain, and emergence delirium compared with control groups receiving care as usual [17]. Similarly, children often fear and apprehend dental visits. Researchers are investigating the use of IVR as a familiarization tool before dental visits to improve the experience of children. It is expected that by using a VR tool, children will become less fearful by becoming more familiar with what to expect, which in turn can decrease the number of missed dental visits [44]. As demonstrated by most up-to-date breakthrough studies, VR game preparation is a promising venue and this ideology resonates with the aim of this study, which is to broaden and confirm the beneficial use of VR game preparation in anxiety-related MRI procedures.

### Clinical Trial

Our study’s field test phase followed by a larger RCT aims to transpose and demonstrate the beneficial effect of VR game preparation for MRI procedures because its use has already been shown to improve patient satisfaction in other anxiety-related medical procedures (as previously mentioned). To our knowledge, no algorithm has been developed to identify children who are more likely to be responsive to VR intervention. The study team aims to develop this innovative biofeedback algorithm through data collection and analysis. This is a scientific breakthrough, in that the development of such an algorithm can potentially identify modifiable participant factors that future research studies can address to potentialize future use of VR interventions in the medical field. Furthermore, this project will provide evidence-based knowledge on a nonpharmacological method for anxiety management for patients required to undergo MRI studies through an innovative intervention. Our study provides an alternative method to manage anxiety and better prepare patients for an awake MRI procedure. The preprocedural IVR game preparation can potentially decrease the need for sedation by educating the child about the MRI procedure. The biofeedback will also help predict which children are more responsive to this type of intervention. It is expected that the experimental group, as demonstrated in other studies, will experience less procedural anxiety after VR game exposure, which will be featured in this study with analogous physiological parameters (eg, slower heart rate), and a smaller number of terminated MRI studies as increased patient collaboration will reduce anxiety-related movement artifacts. Positive participant and health care worker satisfaction levels will also encourage the use of VR game preparation in other anxiety-related medical procedures in the future. As opposed to physically bulky replica scanners that can be used only on the day of the procedure to help prepare children for MRI studies, our intervention is more versatile and user friendly. As VR technology is becoming more accessible to the public, our VR game preparation can be experienced using portable VR goggles at home before the day of the MRI procedure. As most patients requiring an MRI examination come from an outpatient setting, the portability of our intervention requires less on-site time and staff to organize and operate the replica scanner on the day of the imaging study. Thus, the VR game preparation that our team developed can increase patient turnover in the radiology department, shorten the waitlist for other patients requiring MRI examinations, and facilitate medical access. Participants will have an increased comprehension of the MRI study, and this positive association with medical equipment can further benefit and improve children’s experience with hospital settings.

### Benefits for Study Participants

We strongly believe that IVR is an innovative intervention that could be offered to most of the children scheduled for a medical imaging procedure. For children eligible to use VR, and their family, it provides a more immersive preparation and could decrease their anxiety by exposing them to a better preparation to the intervention ahead of time. It is an easy-to-use intervention that could be set up in a few minutes and that does not require an additional resource to make it work.

Preprocedural IVR game preparation may help reduce MRI examination–related procedural anxiety in children receiving care, increase satisfaction rate of families whose children receive care and reduce intervention time or repeats required for MRI studies. As for medium- to long-term impact, there is reduced fear in the hospital environment, reduced anticipation of the recurring imaging study procedures, and improved quality of care for children.

### Challenges and Limitations

Recruitment of the necessary sample size of patients may be difficult to estimate because cases requiring MRI examinations happen randomly. Furthermore, the game used during this study was designed by the intervention development team to be engaging for our target audience, to be easy to use within the parameters necessary to the completion of the study, and to minimize cybersickness. As a result, conclusions from this study may be difficult to extrapolate outside of our own controlled setting and may need the same game or another one designed with the same parameters in mind.

## Appendix

Multimedia Appendix 1 French-Canadian version of the State-Trait Anxiety Inventory for Children (STAIC-F).

Multimedia Appendix 2 Children’s Fear Scale.

## Abbreviations

ANCOVA: analysis of covariance
CFS: Children’s Fear Scale
CONSORT: Consolidated Standards of Reporting Trials
GRS: Graphic Rating Scale
IVR: immersive virtual reality
MRI: magnetic resonance imaging
PedIMMPACT: Pediatric Initiative on Methods, Measurement, and Pain Assessment in Clinical Trials
RCT: randomized controlled trial
REDCap: Research Electronic Data Capture
STAIC: State-Trait Anxiety Inventory for Children
STAIC-F: State-Trait Anxiety Inventory for Children, French-Canadian version
VR: virtual reality
