# Supplementary material for: The Efficacy of Virtual Reality Game Preparation for Children Scheduled for Magnetic Resonance Imaging Procedures (IMAGINE): Protocol for a Randomized Controlled Trial
Source: JMIR Res Protoc. 2022 Jun 13;11(6):e30616. doi: 10.2196/30616 (PMC9237773; doi:10.2196/30616)
Supplement: Multimedia Appendix 1 [file resprot_v11i6e30616_app1.docx]

**Appendix 1**

**Inventaire d’anxiété situationnelle et de trait pour enfants**

**DIRECTIVES**

Tu trouveras ci-dessous des phrases que les garçons et les filles utilisent pour se décrire. Lis chaque phrase attentivement et décide comment tu te sens ***maintenant****.* Ensuite*,* place un X dans le carré devant le mot ou la phrase qui décrit le mieux comment tu te sens. Il n’y a pas de bonne ou de mauvaise réponse. Ne perds pas trop de temps sur une phrase. Souviens-toi que tu dois essayer de trouver le mot ou la phrase qui décrit le mieux comment tu te sens maintenant, ***à ce moment-ci***.

1. **Je me sens .............**  très calme calme pas du tout calme

2.  **Je me sens .............** très bouleversé-e bouleversé-e pas bouleversé-e

1. **Je me sens .............** très aimable aimable pas aimable

4. **Je me sens .............**  très nerveux-se nerveux-se pas nerveux-se

5. **Je me sens .............**  très agité-e agité-e pas agité-e

6. **Je me sens .............**  très reposé-e reposé-e pas reposé-e

7. **Je ressens .............** beaucoup de peur un peu de peur pas de peur

8. **Je me sens .............** très détendu-e détendu-e pas détendu-e

9. **Je me sens .............** très inquiet-ète inquiet-ète pas inquiet-ète

10. **Je me sens .............**  très satisfait-e satisfait-e pas satisfait-e

11. **Je me sens .............** très effrayé-e effrayé-e pas effrayé-e

12. **Je me sens .............** très heureux-se heureux-se pas heureux-se

13. **Je me sens .............** très sûr-e sûr-e pas sûr-e

14. **Je me sens .............** très bien bien pas bien

15. **Je me sens .............**  très troublé-e troublé-e pas troublé-e

16. **Je me sens .............** très tracassé-e tracassé-e pas tracassé-e

17. **Je me sens .............** très gentil-le gentil-le pas gentil-le

18. **Je me sens .............**  très terrifié-e terrifié-e pas terrifié-e

19. **Je me sens .............**  très mêlé-e mêlé-e pas mêlé-e

20. **Je me sens .............**  très enjoué-e enjoué-e pas enjoué-e

**DIRECTIVES**

Tu trouveras ci-dessous des phrases que les garçons et les filles utilisent pour se décrire. Lis attentivement chaque phrase et décide comment tu te sens ***en général***. Ensuite, place un X dans le carré devant la réponse qui semble te décrire le mieux. Il n’y a pas de bonne ou de mauvaise réponse. Ne perds pas trop de temps sur une phrase. Souviens-toi de choisir le mot qui décrit le mieux comment tu te sens ***habituellement***.

1. Je suis préoccupé-e par l’idée de faire Presque jamais Quelquefois Souvent

des erreurs

2. J’ai envie de pleurer Presque jamais Quelquefois Souvent

1. Je me sens malheureux-se Presque jamais Quelquefois Souvent
2. J’ai de la difficulté à prendre des Presque jamais Quelquefois Souvent

décisions

5. Il est difficile pour moi de faire face à Presque jamais Quelquefois Souvent

mes problèmes

6. Je m’inquiète trop Presque jamais Quelquefois Souvent

7. Je deviens bouleversé-e quand je Presque jamais Quelquefois Souvent

suis à la maison

8. Je suis gêné-e Presque jamais Quelquefois Souvent

9. Je me sens troublé-e Presque jamais Quelquefois Souvent

10. Des idées sans importance me Presque jamais Quelquefois Souvent

passent par la tête et me tracassent

11. Je m’inquiète à propos de l’école Presque jamais Quelquefois Souvent

12. J’ai de la difficulté à décider quoi Presque jamais Quelquefois Souvent

faire

13. Je remarque que mon cœur bat vite Presque jamais Quelquefois Souvent

14. J’ai peur et je n’en parle à personne Presque jamais Quelquefois Souvent

15. Je m’inquiète pour mes parents Presque jamais Quelquefois Souvent

16. Mes mains sont moites (mouillées) Presque jamais Quelquefois Souvent

17. Je m’inquiète à propos de choses Presque jamais Quelquefois Souvent

qui pourraient arriver

18. J’ai de la difficulté à m’endormir le soir Presque jamais Quelquefois Souvent

19. J’ai une sensation bizarre dans Presque jamais Quelquefois Souvent

mon estomac

20. Je m’inquiète à propos de ce que les Presque jamais Quelquefois Souvent

autres pensent de moi

Référence originale :

Spielberger, C. D., Edwards, C D., Lushene, R. E., Montuori, J., Platzek, D. (1973). STAIC Preliminary Manual for the State-Trait Inventory for Children («How I Feel Questionnaire»). Palo Alto, CA: Consulting Psychologists Press Inc.

© Tous droits réservés 1970 C.D. Spielberger

Traduction par Janel Gauthier et Stéphane Bouchard, Université Laval.

Adapté par Lyse Turgeon (1998), Centre de recherche Fernand-Seguin, Hôpital Louis-H. Lafontaine.
