# Supplementary material for: The Efficacy of Virtual Reality Game Preparation for Children Scheduled for Magnetic Resonance Imaging Procedures (IMAGINE): Protocol for a Randomized Controlled Trial
Source: JMIR Res Protoc. 2022 Jun 13;11(6):e30616. doi: 10.2196/30616 (PMC9237773; doi:10.2196/30616)
Supplement: Multimedia Appendix 2 [file resprot_v11i6e30616_app2.docx]

**Appendix 2**

**Child Fear Scale**

**ANXIÉTÉ/ PEUR :**

*
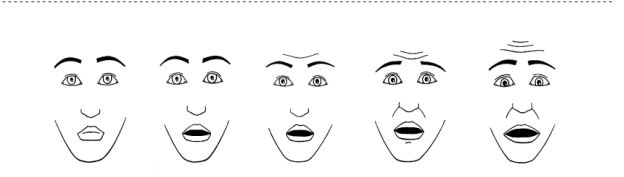
*

**Peur/Anxiété ressentie:**

CFS : ______/4 (auto-évaluation)

*Instruction : Ces visages représentent les différents niveaux de peur qu’il est possible de ressentir. Le premier visage (celui à l’extrémité gauche) n'a pas peur du tout, le second visage a un peu plus peur, le troisième a encore plus peur et le dernier visage (celui à l’extrémité droite) ressent la plus grande peur possible. Regarde ces 5 visages et choisis celui qui démontre le plus la peur que tu ressens face à la procédure qui s’en vient.*
